# Molecular cloning, prokaryotic expression and its application potential evaluation of interferon (IFN)-ω of forest musk deer

**DOI:** 10.1038/s41598-023-37437-x

**Published:** 2023-06-30

**Authors:** Xi Wu, Wei Yang, Jian-guo Cheng, Yan Luo, Wen-long Fu, Lei Zhou, Jie Wu, Yin Wang, Zhi-jun Zhong, Ze-xiao Yang, Xue-ping Yao, Mei-shen Ren, Yi-meng Li, Jie Liu, Hui Ding, Jia-nan Chen

**Affiliations:** 1 College of Veterinary Medicine, Sichuan Agricultural University, Wenjiang, 611130 Sichuan Province China; 2 Sichuan Institute of Musk Deer Breeding, Dujiangyan, 611830 Sichuan Province China

**Keywords:** Immunology, Molecular biology

## Abstract

Forest musk deer (*Moschus berezovskii*) are currently a threatened species under conservation, and the development of captive populations is restricted by health problems. To evaluate the application potential of interferon (IFN)-ω in the prevention and control of forest musk deer disease, 5 forest musk deer IFN-ω (fmdIFNω) gene sequences were successfully obtained by homologous cloning method for the first time. FmdIFNω5 was selected and recombinant fmdIFNω protein (rIFNω) was successfully expressed by pGEX-6P-1 plasmid and *E. coli* expression system. The obtained protein was used to stimulate forest musk deer lung fibroblasts cells FMD-C1 to determine its regulatory effect on interferon-stimulated genes (ISGs). In addition, an indirect ELISA method based on anti-rIFNω serum was established to detect endogenous IFN-ω levels in 8 forest musk deer. The results showed that there were 18 amino acid differences among the 5 fmdIFNω subtypes, all of which had the basic structure to exert the activity of type I IFN and were close to *Cervus elaphus* IFN-ω in the phylogenetic tree. The protein expressed was 48 kDa, and the transcription levels of all ISGs were increased in FMD-C1 cells stimulated by rIFNω, and the amount of transcription accumulation was time-dependent. Meanwhile, Anti-rIFNω serum of mice could react with both rIFNω and forest musk deer serum, and the OD_450nm_ value of forest musk deer serum with the most obvious symptoms was the highest, suggesting that the level of natural IFN-ω in different forest musk deer could be monitored by the rIFNω-based ELISA method. These results indicate that fmdIFNω has the potential as an antiviral drug and an early indication of innate immunity, which is of great significance for the prevention and control of forest musk deer diseases.

## Introduction

Forest musk deer (*Moschus berezovskii*) is the largest species of musk deer and mainly distributed in the high altitude mountains of China and Vietnam^1^. Musk, which has a remarkably high economic and medicinal value, is secreted by the musk gland located in the groin of male forest musk deer^2,3^. Caused by over-exploitation, shrinkage in distribution, habitat destruction and degradation, the wild forest musk deer resources are on the verge of extinction. So it was classified a class I-protected animal in China and it had been assessed for The IUCN Red List of Threatened Species in 2015^4,5^. In order to solve the contradiction between supply and demand of musk and realize the protection of forest musk deer resources, the artificial breeding industry of musk deer has been launched over 60 years in China. However, due to the short domestication history, immature pathogen detection technology, and limited means of disease control, the mortality rate of captive forest musk deer is relatively high, and the population growth is slow^6–8^. Interferon (IFN) is a secretory glycoprotein stimulated by virus and other heterologous substances, with the functions of regulating immune function, broad spectrum antiviral and inhibiting cell proliferation^9,10^. It is of great significance to study the IFN of forest musk deer for the development of artificial breeding industry of them.

According to their differences of genetic, structural and functional characteristics and cell surface receptors, IFNs are divided into type I, II and III classes, and their mediated innate immune response together forms a front-line of cell-autonomous defense against pathogens^11,12^. IFN-ω belongs to type I IFNs, which was found in humans about 30 years ago and is mainly produced in white blood cells^9,13,14^. When virus invasion induces the body to produce anti-infection mechanism, about 15% of the antiviral effect is caused by IFN-ω completed^15^. As the same as type I IFNs, IFN-ω is released in the process of innate immune activation, and does not play a role in itself, but relies on activating downstream effectors to achieve antiviral and innate immune regulation functions^16^. ISGs among them could provide adequate cellular immunity against positive-, negative-, and double-stranded RNA viruses, DNA viruses, and even intracellular bacteria and parasites^17^. Studies showed that the main antiviral proteins induced by IFN-ω included *Mx1*, *ISG15*, *IFIT3* and *ISG56*, which play important roles in the inhibition of the viral life cycle, from entry, replication, assembly to release^6,17–19^.

Except that IFN-ω gene is not found in *Canis familiaris* and *Mus musculus*, IFN-ω has been identified in common mammals, in which there is only 1 IFN-ω subtype in *Homo sapiens* while up to 25 in *Bos indicus*^18,20–22^. As an antiviral drug, IFN-ω is a good substitute for IFN-α and is widely used in patients with chronic human hepatitis C virus infection (HCV)^23^. As an immunomodulator, IFN-ω has been registered in many countries for canine parvovirus (CPV), feline leukemia virus (FeLV), and feline immunodeficiency virus (FIV) infections^19,24–27^. As an anti-tumor drug, it can be used in the treatment of canine and feline breast cancer, and has shown a certain therapeutic effect on fibrosarcoma^28,29^. Additionally, anti-IFN-ω antibody has become an important diagnostic tool for human autoimmune polyglandular syndrome type 1 (APS-1)^30,31^. However, there is no research on fmdIFNω has been published, so in this field, a broad space is still for exploration. In this study, the cloning and bioinformatics analysis of fmdIFNω were carried out and the prokaryotic expression system was used to express it externally. Besides, the application potential of fmdIFNω was briefly evaluated, which provided the relevant theoretical basis for the application of fmdIFNω, and also provided help for the protection of forest musk deer resources and the development of artificial musk deer industry.

## Results

### Cloning, subtypes classification and predicted characteristics of fmdIFNω

**Figure 1:**
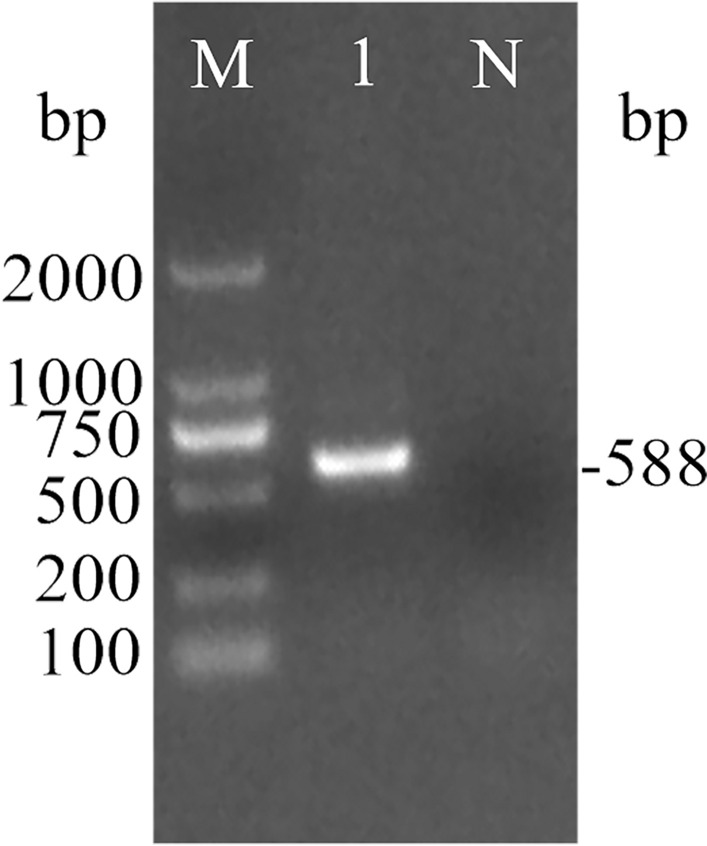
Gel electrophoresis of fmdIFNω amplified by PCR. Note: M, DL2000 marker; 1, positive; N, negative control.

Using cDNA from peripheral blood lymphocytes of forest musk deer as template and IFNω as primers, the gel electrophoresis results of fmdIFNω are shown in Fig. 1. A specific band can be seen at about 588 bp on the lane 1, which met the expected size of the product.

**Figure 2:**
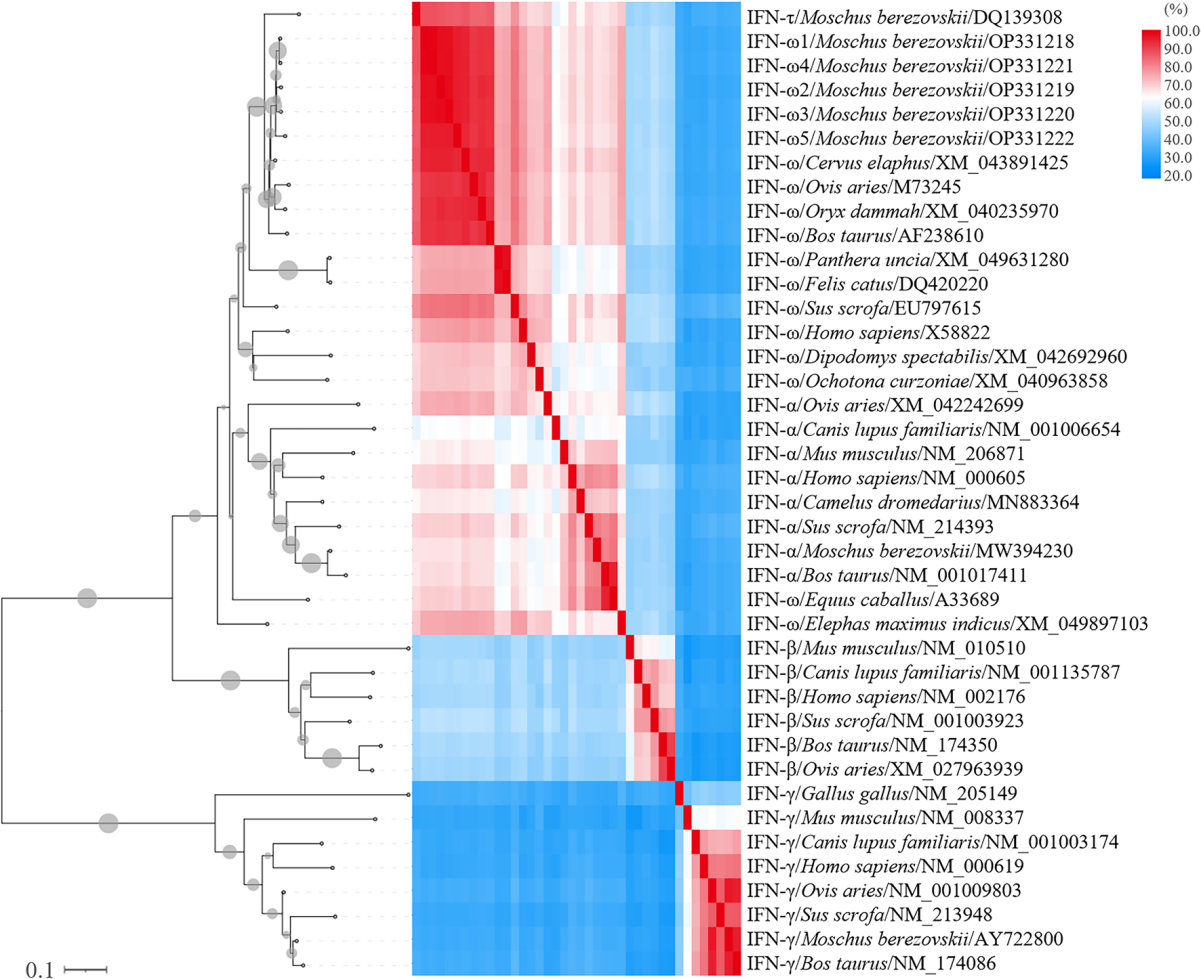
Phylogenetic reconstruction and nucleotide homology matrix of fmdIFNω. The phylogenetic tree generated using neighbor-joining method (1000 bootstrap replicates). The dot size of each branch represents the confidence score, and the label describes the IFN subtype, the latin name of species, and their Genbank accession number. The horizontal labels of the homology matrix are in the same order as the vertical.

The purified product was ligated with pMD19-T and transformed into competent *E. coil* DH5α. By sequencing the positive clones, 5 IFN-ω subtypes of forest musk deer were obtained, which were named fmdIFNω1-fmdIFNω5, and their nucleotide sequences were all 588 bp length and encoded 195 amino acids. Phylogenetic tree and homology matrix were constructed for the nucleotide sequences of fmdIFNω, and the results were shown in Fig. 2. The 5 fmdIFNω subtypes clustered in the phylogenetic tree, and were in the same branch as IFN-ω of ruminants such as *Bos taurus*, *Cervus elaphus*, *Ovis aries* and *Oryx dammah*, with a confidence of 99. Among the 5 subtypes, nucleoacid sequence homology was 95.9–99.5%, and it was 92.2–95.2% homology with other ruminants. Compared with IFN-τ, IFN-α, IFN-β and IFN-γ, the highest homology value was 88.3%, 76.4%, 53.9% and 35.7%, respectively. In the reconstructed phylogenetic tree, *Cervus elaphus* IFN-ω was the most closely related to fmdIFNω especially fmdIFNω5, which belongs to a small independent clade. In view of the high homology of the 5 subtypes, the subsequent structural prediction results were all represented by fmdIFNω5.

**Figure 3:**
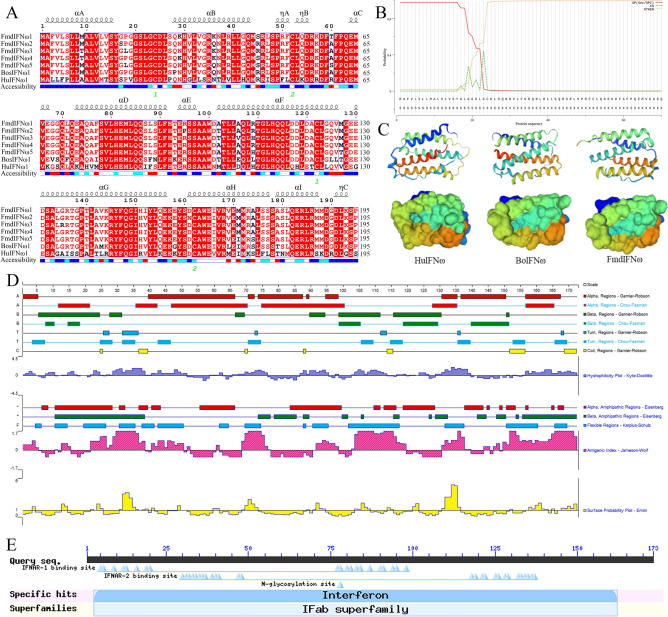
Predicted and compared structural characteristics of fmdIFNω. (**A**) Homology of amino acid sequence of each subtype of IFNω. Note: 1 and 2 are conserved cysteine residues linked. (**B**) Signal peptide prediction of fmdIFNω. (**C**) Comparison of tertiary structure of IFNω. (**D**) Secondary structure, hydrophilicity, ampipathic regions, antigenicity and surface probability analysis of fmdIFNω. (**E**) Domain and glycosylation site prediction of fmdIFNω.

The predicted and compared structural characteristics of fmdIFNω are shown in Fig. 3. The sequence is a secreted protein, with 1-23 amino acids as the signal peptide fragment, which is consistent with the signal peptide site of most type I IFNs, and 24-172 amino acids as the mature peptide of the sequence (Fig. 3B). The physicochemical properties of the 5 fmdIFNω mature peptides were predicted (Table 1). The results showed that the molecular weight was about 19.5 kDa and the formulas of the 5 subtypes were not completely same. It is worth noting that the Estimated half-life of fmdIFNω in mammalian reticulocyte in vitro is only 1.2 h, and the instability indexes are between 58.42 and 63.15, indicating that they are unstable proteins. Others, the theoretical PI, total number of negatively charged residues (Asp + Glu), total number of positively charged residues (Arg + Lys), extinction coefficients, aliphatic index and grand average of hydropathicity are not very different overall. The amino acid alignment of the 5 fmdIFNω sequences revealed 18 different coding amino acids (Fig. 3A). Considered with bovine (BoIFNω1) and human (HuIFNω1), it showed that there were 4 conserved cysteine residues at positions 1, 29, 99, and 139, 1 conserved arginine residue at position 161, and 3 conserved proline residues at positions 26, 39, and 116 in the mature peptide. The predicted secondary structure showed that fmdIFNω was mainly composed of α-helix and random coil. The tertiary structure was highly similar to that of huIFNω and boIFNω, including 5 major α-helical structures (Fig. 3C). The conserved domain of fmdIFNω was predicted, and the results showed that it was aligned as IFN, a member of the IFN-α/β superfamily, and 35-152 amino acid sequence were conserved domain sites of the protein. The mature peptide sequence also contained binding sites for IFNAR-1 and IFNAR-2. The IFNAR-1 binding sites were located at positions 5-20 and 77-98, while the latter were located at positions 30-48 and 117-136. The results also showed that there was an *N*-glycosylation site at position 78 of fmdIFNω mature peptide sequence (Fig. 3E).

**Table 1:** Physicochemical properties of deduced fmdIFNω proteins.

Proteins	Formula	Molecular weight	Teoretical PI	Asp + Glu	Arg + Lys	Extinction coefficients (280 nm)	Estimated half-life	Instability index	Aliphatic index	Grand average of hydropathicity
FmdIFNω1	C_848_H_1360_N_246_O_260_S_12_	19,546.34	5.73	23	19	15,720/M^−1^ cm^−1^	1.2 h (mammalian reticulocytes, in vitro) > 20 h (yeast, in vivo) > 10 h (*E. coli*, in vivo)	63.15	86.22	− 0.355
FmdIFNω2	C_846_H_1351_N_247_O_260_S_12_	19,527.25	5.52	22	17			58.42	85.64	− 0.357
FmdIFNω3	C_854_H_1367_N_251_O_260_S_12_	19,695.49	5.86	23	19			60.67	86.74	− 0.378
FmdIFNω4	C_847_H_1358_N_246_O_259_S_12_	19,516.31	5.73	23	19			62.43	86.80	− 0.341
FmdIFNω5	C_842_H_1339_N_245_O_264_S_10_	19,438.98	5.30	23	16			60.40	87.33	− 0.360

### Prokaryotic expression, purification, and confirmation of rIFNω

**Figure 4:**
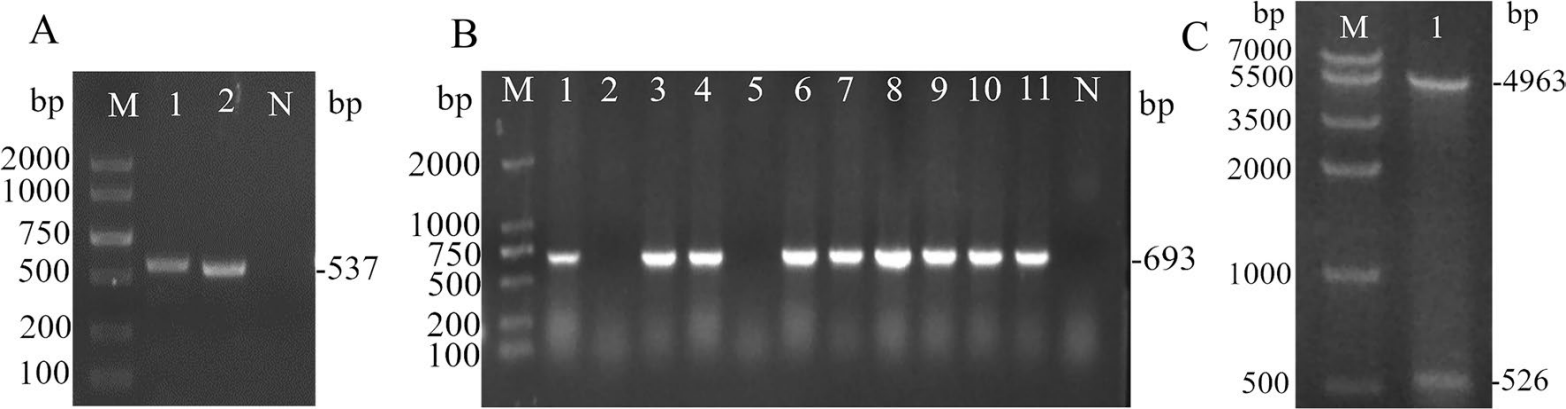
Gel electrophoresis by PCR. (**A**) Amplification results by ExIFNω primers. Note: 1, 2: positive. (**B**) Amplification results by pGEX-6P-1 primers. Note: 1, 3–4, 6–11: positive transformants; 2, 5: negative transformants. (**C**) Double enzyme digestion results of pIFNω.

The extracted pMD19-T-IFNω plasmid was used as the template and ExIFNω-F/R used as primer for PCR amplification. The results showed a specific band at about 537 bp on the lane 1 and 2, which was in line with the expected size of the product (Fig. 4A). pIFNω was transformed into *E.coli* BL21, and colonies were selected for identification. The PCR identification results were shown in Fig. 4B, and 9 out of 11 colonies appeared bands at about 693 bp, which were positive transformants. The results of double enzyme digestion showed that pIFNω was completely dissected, and two bands appeared at 4963 bp and 526 bp, corresponding to line plasmid pGEX-6P-1 and IFNω, respectively, indicating that the expression vector pIFNω was successfully constructed (Fig. 4C). Sequencing results showed that the sequence was 100% identical with fmdIFNω5, which could be used for subsequent experiment.

**Figure 5:**
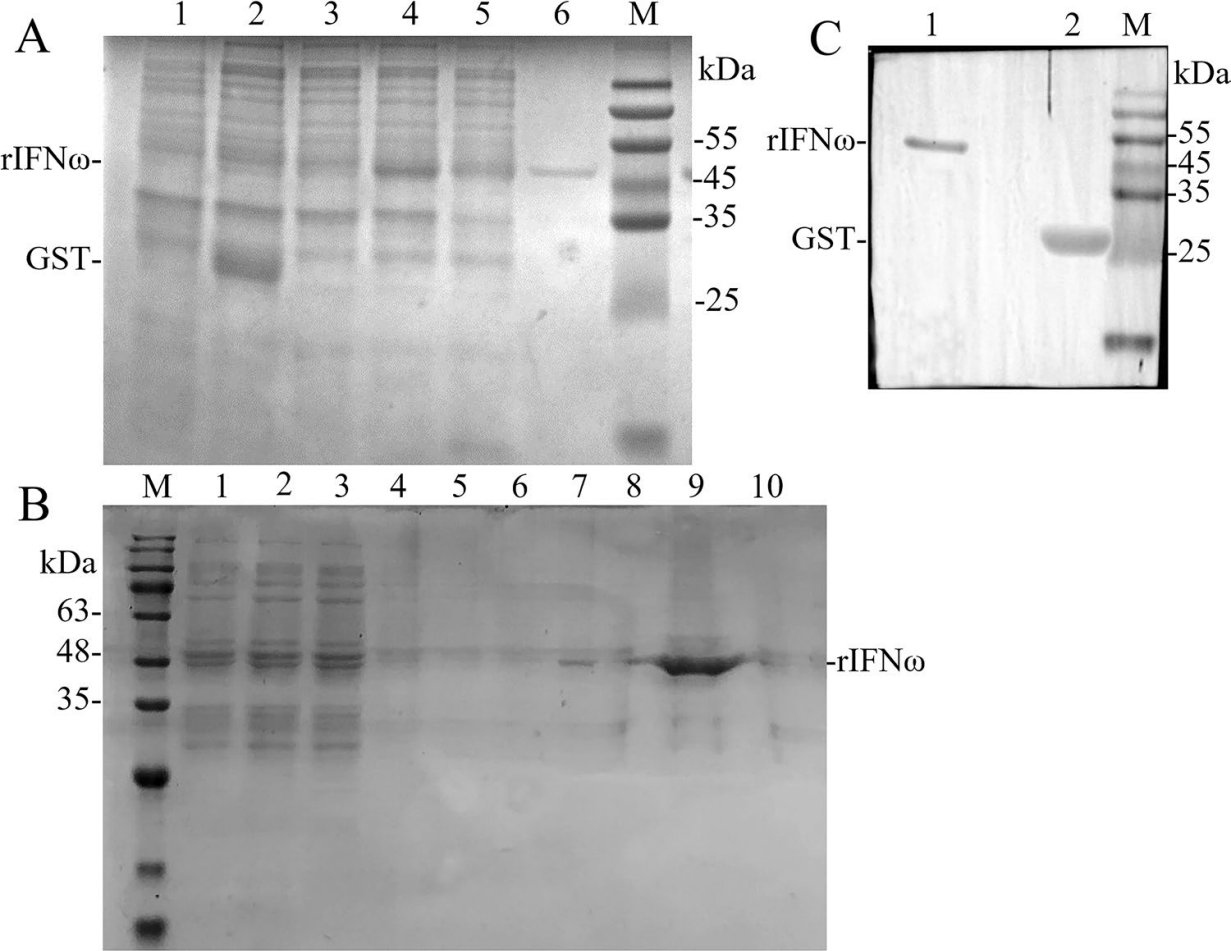
Expression, purification and confirmation of rIFNω proteins from *E. coli* BL21. (**A**) SDS-PAGE results of preliminary induction of rIFNω expression. Note: M, protein molecular weight standard (15–120 kDa); 1, uninduced bacteria with untreated pGEX-6P-1; 2, induced bacteria with untreated pGEX-6P-1; 3, uninduced rBL21; 4, induced rBL21; 5, the supernatant of induced rBL21 after ultrasonic crushing; 6, precipitation of induced rBL21 after ultrasonic crushing. (**B**) Purification result of rIFNω. Note: M, protein molecular weight standard (11–180 kDa); 1–3, flow through liquid; 4–5, rinse liquid; 6–8, eluent; 9, ultrafiltration concentrate rIFNω; 10, filtrate. (**C**) Western blot confirmation. Note: 1, purified rIFNω; 2, induced bacteria with untreated pGEX-6P-1. M, Protein molecular weight standard (15–120 kDa).

SDS-PAGE results of preliminary induction of proteins expression are shown in Fig. 5A. An obvious protein band at about 26 kDa was found in bacteria with untreated pGEX-6P-1 (lane 2) after induction, while no obvious band was found in other lanes. At the same time, the induced rBL21 (lane 4) showed a band at about 48 kDa, but no obvious band was found in bacteria with untreated pGEX-6P-1 and uninduced rBL21 (lane 1–3). By comparing the gray levels of protein bands in lane 5 and 6, it was concluded that rIFNω could be expressed in both supernatant and precipitation.

Purification of prokaryotic expression products is shown in Fig. 5B. Lanes 1–3 are proteins flow through fluid, and there were still bands existed at about 48 kDa, indicating rIFNω overload. There was no obvious protein band in the rinse solution of lane 4 and 5, indicating that the impurity protein in the purification column had been removed, and only the target protein with specific binding was kept. In lane 7, a single protein band appeared at 48 kDa, and the protein concentration increased after ultrafiltration (lane 9), and there was no obvious band in lane 10, indicating that the purified rIFNω protein was successfully obtained, and the purification effect was good. The concentration of concentrated rIFNω protein was determined by Nano Drop 2000, and it was 0.8 mg/mL.

The results of western blot showed that there were two obvious bands on the PVDF membrane after ECL development, respectively located near 48 kDa and 26 kDa. According to the loading proteins and the use of antibody, the two bands are rIFNω and GST-tag respectively, which was in line with the experimental expectation (Fig. 5C).

### Detection of the regulatory activity of rfmdIFNω on ISG transcription

According to the established standard curve of *IFIT3* primers, the amplification efficiency was 90.2%,* r*^*2*^ was 0.997, the slope was − 3.582, and the melting curve showed a single peak, which indicating the specificity and the amplification efficiency of *IFIT3* primer pairs meet the requirement for qPCR.

**Figure 6:**
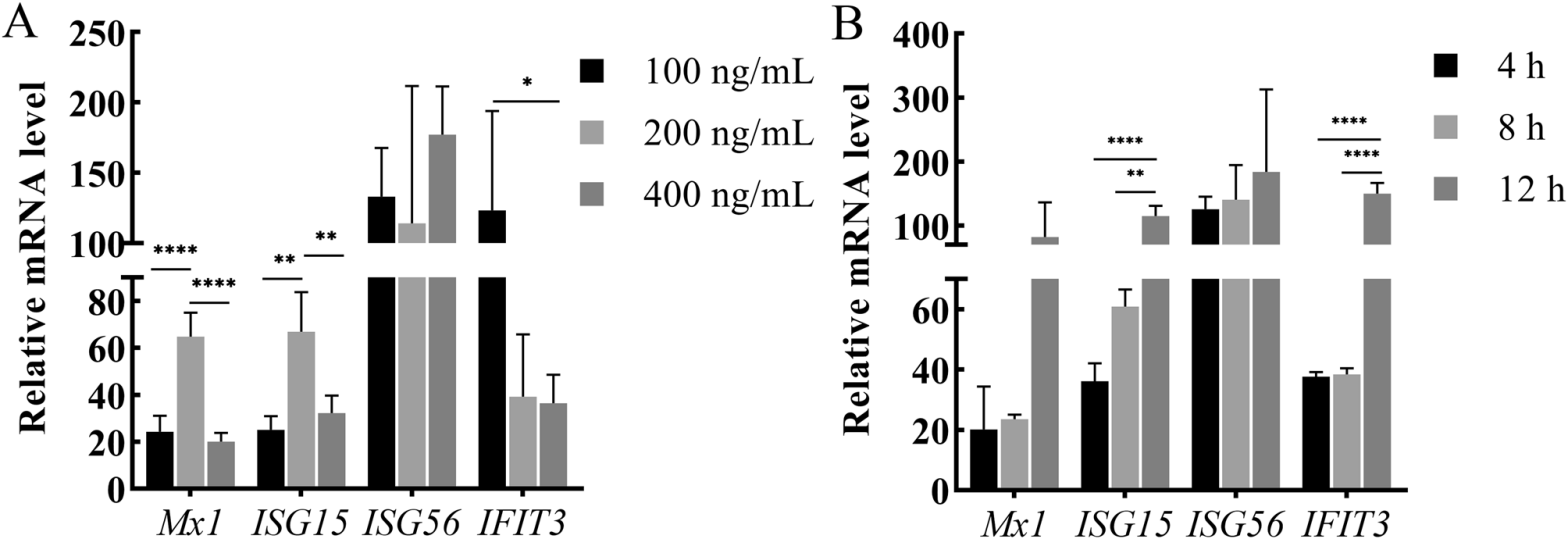
Regulation results of rIFNω on ISGs transcription. (**A**) Dose-dependent results. (**B**) Time-dependent results. *: P < 0.05.

After rIFNω stimulation, the ISGs transcription results of FMD-C1 cells were shown in Fig. 6. The transcriptional levels of *Mx1*, *ISG15*, *ISG56* and *IFIT3* did not show concentration dependence. Within 6 h, the transcriptional levels of *Mx1* and *ISG15* reached the highest when the concentration of rIFNω was 200 ng/mL. *ISG56* was the highest at the concentration of 400 ng/mL, while *IFIT3* was at the concentration of 100 ng/mL. According to the time-dependent results, the transcript accumulation of *Mx1*, *ISG15*, *ISG56* and *IFIT3* all reached the maximum at 12 h. In general, the transcriptional levels of ISGs were all increased at the concentration of 100–400 ng/mL at 4–12 h after stimulation, with the lowest relative expression level of about 20 times and the highest relative expression level of 217 times, indicating that rIFNω had the activity of regulating ISGs transcription in FMD-C1 cells.

### Optimization and application of rIFNω-based indirect ELISA

**Figure 7:**
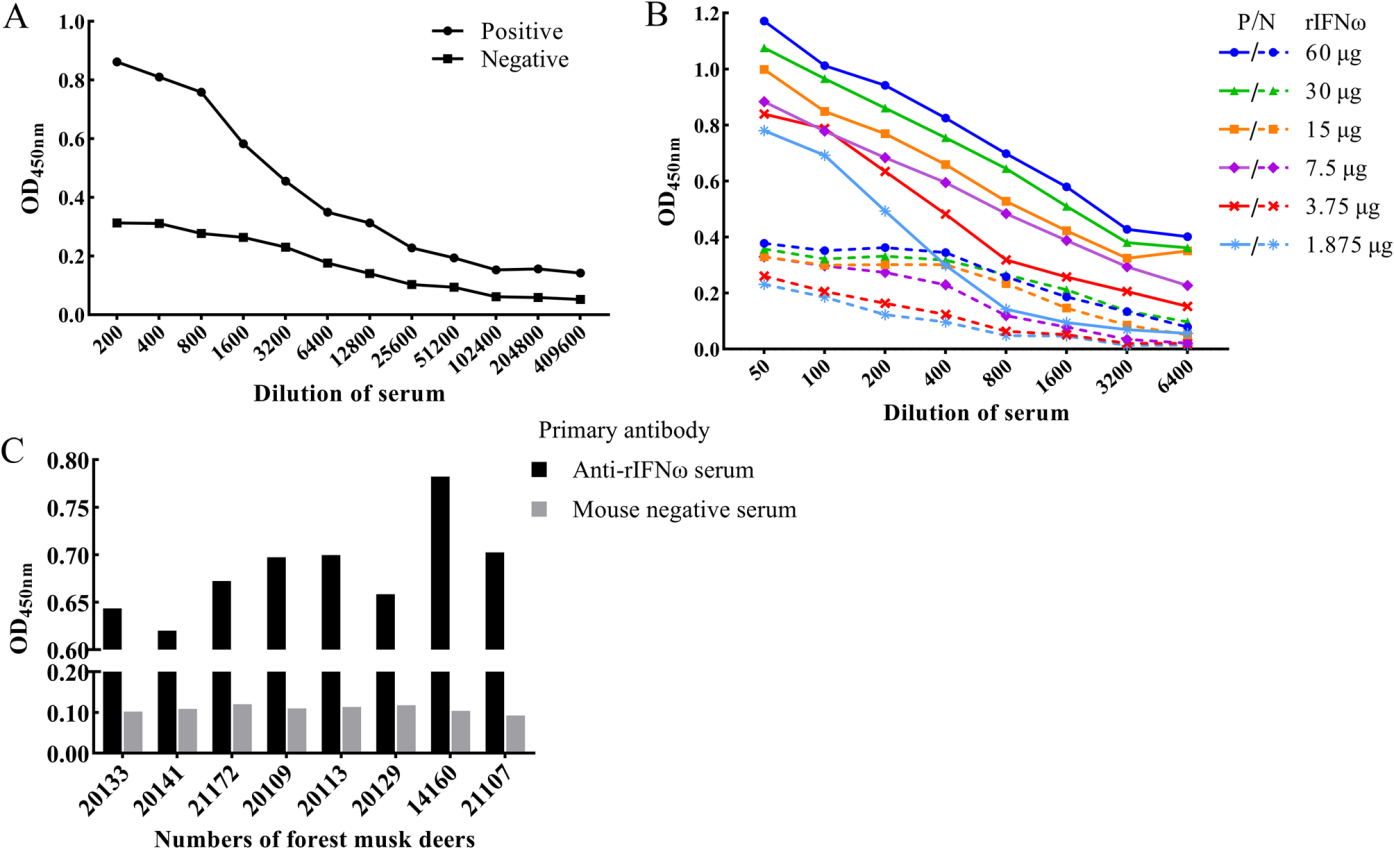
Optimization and application of rIFNω-based indirect ELISA. (**A**) Serum antibody titer detection in mouse. (**B**) Reactivity of rIFNω protein at different concentrations with different dilutions of positive and negative anti-rIFNω sera determined using a checkerboard titration. (**C**) Natural IFN-ω detection in sera of 8 forest musk deer by the rIFNω-based indirect ELISA.

The results of serum polyclonal antibody titer detection in mice were shown in Fig. 7A. The highest dilution multiple of the serum, which was more than 2.1 times the OD_450 nm_ value of negative serum, was taken as the criterion for judging serum titer. According to it, the polyclonal antibody titer of mouse serum was 1:1600. The checkerboard titration results showed that when the positive OD_450 nm_ value was closest to 1, the maximum P/N value was 3.105. Under this condition, the optimal working concentration of rIFNω was 60 μg/mL, and the optimal serum dilution was 1:50 (Fig. 7B). Natural IFNω detection results in sera of 8 forest musk deer by the rIFNω-based indirect ELISA was shown in Fig. 7C. The OD_450 nm_ values of the 8 blood samples were all higher than 2.1 compared with the negative. The highest OD_450 nm_ value was 0.796, indicating a relatively high content of natural IFN-ω in vivo. The forest musk deer was numbered 14160 and had obvious symptoms of disease.

## Discussion

Dispite the type I IFNs bind to the same heterodimeric receptor, the subtypes was shown to have different potencies in various experimental systems^32^. The causes may be that the differences in promoter sequences and biochemical properties contribute to distinct functional activities^16,33^. Therefore, the gene cloning and protein expression of fmdIFNω are irreplaceable for the study of its mechanism and application. In this study, the nucleotide sequences of the cloned 5 fmdIFNω subtypes are with high homology. Although there are 18 amino acid discordations, both cysteine and proline sites are all consistent with known mammalian IFN-ω. These conserved amino acid residues are involved in the formation of protein spatial structures, thus partly determining the similarity of tertiary structures.

Glycosylation affects protein solubility and stability, and has an important effect on IFN activity. Studies have shown that glycosylated IFN-ω can induce sterol regulatory element binding transcription factors, activate enhancer binding protein 2-like YY1 site, IFN consensus sequence and conserved sequence binding protein, etc.^34^. For bovine viral diarrhea virus and yellow fever virus, glycosylated IFN-ω showed a stronger effect^35^. However, 14 of the 23 bovine IFN-ω subtypes did not have an *N*-glycosylation site, including boIFNω1. Although fmdIFNω showed high similarity to boIFNω1 in the previous study, it was fortunately found an N-glycosylation site was at position 78 of the mature peptide sequence. It indicates greater application potential of the protein. In addition, fmdIFNω also contains binding sites for IFNAR-1 and IFNAR-2. They are transmembrane subunits of IFN-α/β receptor complex. Compared with the IFN-α sequence of forest musk deer, there is only 1 amino acid site forward shift in the IFNAR-2 binding site^36^. The 2 subunits play key roles in receptor identification and biological activity, and their existence ensures that fmdIFNω can induce downstream signal transduction to activate intracellular antiviral and immunomodulatory mechanisms, and exert corresponding biological functions.

Prokaryotic expression system is widely used because of its short expression time, simple method and low cost. BoIFNω3 expressed by *E. coli* system could induce the transcription of downstream genes, protect virus-infected cells and resist cell proliferation^18^. And 2 novel feline IFN-ω proteins expressed by in *E. coli* exhibited effective anti-vesicular stomatitis virus (VSV) activity in Vero et al. cells, showing broad cross-species antiviral activity^37^. These results indicating that it could produce biologically active IFN by prokaryotic expression. Therefore, prokaryotic expression of fmdIFNω protein was selected for preliminary study, and pGEX-6P-1 with GST fusion system was selected as the vector. The mild conditions for protein purification, which is a great advantage of the vector, conducive to keep activity of protein, but high level expression easily make peptides gathered too fast and lead to incorrect insoluble folding form. The decrease of soluble protein is not conducive to the fusion protein specific binding with coupling medium glutathione, therefore, the rate of protein synthesis should be reduced in order to improve the soluble. In this study we chose the condition to express at 16 ℃ for 4 h, and the amount of rIFNω obtained was not much. In addition to the reasons for expressing conditions, due to the differences in tRNA levels between *E. coli* and forest musk deer cells, the translation process may be blocked, affecting translation efficiency and reducing protein expression level as well. Therefore, if large-scale expression is needed in further study, it can be optimized from 2 aspects: replacing synonymous codons expressing host preference and screening protein expression conditions.

The activity of IFN is the key factor to determine whether it can play the physiological function of anti-virus and immune regulation. The results of FMD-C1 stimulating experiment by rIFNω showed that rIFNω could significantly increase the transcription levels of *Mx1*, *ISG15*, *ISG56* and *IFIT3* in forest musk deer lung fibroblasts cells. The natural IFN has a short half-life and cannot produce long-term effect inside body. The main reason is not only the instability of protein, but also the small molecular weight, which is easy to be expelled from the body through the kidney. The amino acid physicochemical properties of fmdIFNω showed that, similar to other type I IFNs, fmdIFNω had a short half-life and an instability index of 60.4, which was considered as an unstable protein as well. The activity of eukaryotic forest musk deer IFN-α have been analyzed by a study, and the results showed that under the same conditions, the expression level of ISG reached the peak at 6 h, and then showed a decreasing trend^36^. However, in the time gradient group of this experiment, the relative expression levels of the 4 ISGs increased with time, and the transcript accumulation was the highest at 12 h. Since the use of fusion proteins has been shown to be a simple and effective method to prolong the half-life of serum^38^, it is speculated that the recombinant protein used in this experiment has a relatively large molecular weight, and under the condition of cell culturing in vitro, the protein is not easy to be cleared by the immune system, and will not be excreted by metabolism, so it could take continuous effect on FMD-C1. Besides, the signaling strength, kinetics, and specificity of regulatory pathways on ISG transcription are modulated at various levels by distinct mechanisms operating in conjunction, both canonical and non-canonical mechanisms is involved. These complex mechanisms affect the quantitative and temporal differences in ISG transcription under specific circumstances^17^. In like manner, after rIFNω stimulation, the relative expression of ISGs increased by 217 times as much as that of the control group, while the downstream ISG relative expression induced by forest musk deer IFN-α of eukaryotic expressed stimulated FMD-C1 for 6 h was nearly 3000 times as much as that of the control group^36^. It can be seen that different IFNs, different expression systems and different tag proteins may affect the transcription of stimulated ISGs to different degrees. Detail studied of rIFNω protein in vivo half-life and the transcriptional regulation of downstream factors in forest musk deer are still needed.

Due to the short acclimation time, easy stress, and high mortality after onset, it is particularly critical to pay attention to the occurrence and development of forest musk deer diseases in the centralized feeding environment. The level of endogenous IFN can reflect the cellular immune status of the body, so timely understanding the changes of the corresponding IFN in the animal body plays an important role in understanding the occurrence and progress of the body's diseases^39^. At present, detection of IFN level by ELISA has been a commonly used method for adjuvant diagnosis and auxiliary treatment. For example, IFN-γ release assay is a common method for early diagnosis of *Mycobacterium tuberculosis* and *Toxoplasma gondii* infections^40,41^. The severity and prognosis of psoriasis development can be assessed by measuring serum IFN-γ levels^42,43^. There are also studies to characterize the early innate immunity of the body by preparing monoclonal antibodies against IFN-α to detect the level of it^44,45^. In this study, an indirect ELISA method for the detection of endogenous IFN-ω in forest musk deer was also preliminarily established through the prepared anti-rIFNω serum from mice. In fact, the immunogenicity of IFN is weak. In human, it has been reported that only 1 of 175 patients who undergoing rIFN-α2b therapy produced antibodies because of treatment for chronic myelogenic leukemia^46^. In canis, feIFNω was administered orally and subcutaneously to treat canine specific dermatitis, no serum antibodies against rIFNω could be detected in any of the dogs^47^. It can be seen from Fig. 7 that when the adjuvant is mixed, the mice immunized with rIFNω with higher molecular weight than the natural IFN. Although the antibody titer obtained is only 1:1600, the induced antibody can specifically bind to rIFNω and the serum of forest musk deer, while the serum of the mice not immunized has no reactivity with both, indicating that rIFNω has certain immunogenicity, the ELISA based on anti-rIFNω serum can be used as a monitoring tool for natural IFN in forest musk deer serum, which confirms that rIFNω maintains the same antigen conformational epitope as natural IFN-ω. Moreover, the forest musk deer with the most obvious disease symptoms detected the highest OD_450nm_ value, indicating that fmdIFNω has the potential as a marker of early innate immunity.

Finally, type I IFN are also crucial mediators of inflammatory processes during bacterial infections. They are able to restrict bacterial infection by inducing ISGs expression in vitro and in some in vivo contexts. Among ISGs there are several components of inflammasomes and antibacterial proteins. In this process, regulators of pattern recognition receptors (PRRs) and regulators of antimicrobial signaling pathways are also involved^48^. The purulent diseases caused by bacteria are frequent and lethal in captive forest musk deer. IFN-ω, as a member of type I IFN, mediates similar regulatory pathways. The rIFNω protein expressed in this study may be useful as a new therapeutic agent to control pyogenic diseases of forest musk deer.

In conclusion, 5 subtypes of fmdIFNω gene have been successfully cloned. These subtypes have the necessary infrastructure to play the role of type I IFN. The soluble rIFNω protein was successfully expressed, which had the activity of inducing downstream ISG transcription, and the anti-rIFNω serum of mice could react with rIFNω and forest musk deer serum at the same time, demonstrating fmdIFNω has potential in developing into a therapeutic drug and auxiliary diagnostic reagent for forest musk deer diseases, which is of great significance for the protection and artificial breeding of it.

## Materials and methods

### Materials

The blood samples of forest musk deer were collected from Sichuan Institute of Musk Deer Breeding (Dujiangyan and Maerkang, China). With the help of veterinarians who have years of experience to take care of forest musk deer, 5 mL bloods were drawn from the hindlimb veins of each forest musk deer during routine examinations and treatment. It was completed in 2 times. For the first time, the blood from 2 adult female forest musk deer was quickly stored in sodium citrate anticoagulant tubes for the separation of peripheral blood lymphocytes. The second collection from 8 forest musk deer, with number of 20133, 20141, 21172, 20109, 20113, 20129, 14160 and 21107, included 2 females and 6 males (even numbers at the end of the number indicate females, while the rest are males). They were further divided into 2 juveniles (number 21-), 5 subadults (number 20-), and 1 adult (number 14-). The 8 forest musk deer had different degrees of runny nose and sneezing at the time of blood collection or earlier, among which the number 14160 had the most obvious symptoms, while the numbers 20133 and 20113 were in the improvement stage of disease treatment. Bloods were collected separately in tubes without additives for separating serum to detect the contents of natural IFN-ω in serum.

The expression plasmid pGEX-6P-1 and the lung fibroblast of forest musk deer named FMD-C1 was obtained from the Animal Quarantine Laboratory, Wenjiang Campus, Sichuan Agricultural University (Chengdu, China). For FMD-C1 culturing, the Australian fetal bovine serum and DMEM medium (Hyclone, USA) were needed. DH5α and BL21 (DE3) competent *E. coli* (TIANGEN, Beijing, China) were used for fmdIFNω gene cloning and expression, respectively. The pMD-19T (Simple) vector, DNA ligation kit, restriction enzymes *Eco*R I and *Not* I were purchased from TaKaRa Biomedical Technology (Japan) Co., Ltd. The PBMCs isolation kit (Bayinlai, Wuhan, China), RNA isolation kit (FORGENE, Chengdu, China) and cDNA Synthesis Kit (Vazyme, Nanjing, China) were used in the gene cloning process. The SDS-PAGE kit, protein molecular weight standard (11–180 kDa), coomassie brilliant blue R-250, ultrafiltration centrifuge tube from Beijing Solarbio Technology Co., Ltd were used in the protein expression process. Protein molecular weight standard (15–120 kDa), TMB Chromogen Solution for ELISA, DNA Gel Extraction Kit and BeyoECL Plus were purchased from Shanghai Beyotime Biotechnology Co., Ltd. The Plasmid Mini-Preps Kit, SYBR Green qPCR Mix, GST 4FF Sefinose (TM) Resin Kit, no protein blocking solution, anti-GST Tag mouse monoclonal antibody and HRP-conjugated rabbit anti-mouse IgG monoclonal antibody were obtained from Sangon Biotech (Shanghai) Co., Ltd. Besides, 6-week-old BALB/c mice purchased from Chengdu Dysso Laboratory Animal Co., Ltd. and the QuickAntibody (2W) adjuvant purchased from Beijing Biodragon Immunotechnologies Co., Ltd were used for rIFNω immunization.

### Primer design

The primers used are shown in Table 2. Gene sequences of forest musk deer closely related species, such as *Bos taurus* and *Caprinae*, were used to align with the published forest musk deer whole-genome shotgun sequences in the national center for biotechnology information (NCBI) database by BLAST to identify the fmdIFNω and *IFIT3* DNA sequences not annotated in their genome. Since there’s no intron in type I IFNs, primers that could amplify entire gene sequence were directly designed using the fragment with the highest IFN-ω similarity. The primer for protein expression called ExIFNω, was designed after removing the signal peptide fragment from the obtained fmdIFNω gene sequence, and the restriction site and protective base were added at the 5' end of the primers. Likewise, *IFIT3* primers were designed in the same way as IFN-ω, but the amplify fragment was between 90 and 250 bp in length. Primers named HPRT1 and YWHAZ were used for qPCR detection of reference genes, while Mx1, ISG15, ISG56 and IFIT3 were used for qPCR detection of ISGs. All primer pairs were designed by primer5.0 software and sent to Sangon Biotech (Shanghai, China) Co., Ltd. for synthesis.

**Table 2:** Primers information.

Primer name	Primer sequence (5′–3′)	*T*a (°C)	Product length (bp)	Source
IFNω	F: ATGGCCTTCGTGCTCTCTCT R: TCAAGGTGATTTCAGGTCTC	58	588	SPDX01018843.1: 3586-4173^a^
ExIFNω	F: CGGAATTCTGTGACCTGTCTCAGAACCATG R: TTGCGGCCGCTCAAGGTGATTTCAGGTCTCCATCCA	58	537	OP331222
pGEX-6P-1	F: GGGCTGGCAAGCCACGTTTGGTG R: CCGGGAGCTGCATGTGTCAGAGG	58	188/693	Reference^50^
HPRT1	F: ATGGCCTTCGTGCTCTCTCT R: TCAAGGTGATTTCAGGTCTC	58	129	Reference^51^
YWHAZ	F: ATGGCCTTCGTGCTCTCTCT R: TCAAGGTGATTTCAGGTCTC	58	135	Reference^51^
Mx1	F: ATGGCCTTCGTGCTCTCTCT R: TCAAGGTGATTTCAGGTCTC	56	172	Reference^36^
ISG15	F: ATGGCCTTCGTGCTCTCTCT R: TCAAGGTGATTTCAGGTCTC	56	79	Reference^36^
ISG56	F: ATGGCCTTCGTGCTCTCTCT R: TCAAGGTGATTTCAGGTCTC	56	142	Reference^36^
IFIT3	F: ATGGCCTTCGTGCTCTCTCT R: TCAAGGTGATTTCAGGTCTC	56	222	SGQJ01001479.1: 1277460-1279380^a^

Note: *Ta*: Annealing temperature, the underlined base sequence is the restriction site and protection base.

^a^Gene full/partial sequences location in the forest musk deer genome.

For qPCR analyse, standard curves of the self-designed *IFIT3* primers were established to confirm that the primers could met the requirement. cDNA samples were ten-fold made into 8 dilution series as the standard substance, and qPCR was performed with it as the template. Each dilution was amplified in triplicate PCR amplifications and plotted as mean values to generate a standard curve. The single threshold mode was used to calculate the cycle threshold (Ct) value based on the threshold crossing point of individual fluorescent trace. According to the software ABI 7500 Software v2.0.1, the melting curve, standard curve, amplification efficiency and the linear correlation between template concentration and Ct value were automatically generated.

### FmdIFNω gene cloning

According to the manufacturer’s procedures of kits, lymphocytes were firstly isolated from the collected blood samples and then cultured with 1 μg/mL concancanin A induced at 37 ℃ for 48 h. Total RNA was extracted from the cells, and complementary DNA (cDNA) was synthesized. Taking it as template and IFNω-F/R as primer, PCR amplification was performed. The PCR products were identified by agarose gel electrophoresis. The target DNA fragments purified by DNA Gel Extraction Kit were ligated with pMD19-T vector and then transformed into the competent *E. coli* DH5α. Transformants were identified by PCR and sent to Sangon Biotech (Shanghai) Co., Ltd. for sequencing.

### Gene sequences analysis

The obtained sequences were performed signal peaks proofreading by Snapgene software. Through the DNAMAN software analyzed, duplicates and pseudogenes were removed, and the remaining genes were aligned and typed. In addition, the ProtParam program of ExPASy was used to analyze the physicochemical properties of the amino acids encoded by fmdIFNω. The BLAST in NCBI was used to predict the conserved domains and SignalP 5.0 Server online website was used to predict the signal peptides and transmembrane regions. Moreover, the secondary and tertiary structure of fmdIFNω protein was predicted by PSIPRED and SWISS-MODEL program respectively. Finally, the phylogenetic tree was constructed by MEGA 7.0 software.

### Prokaryotic expression vector construction

FmdIFNω5 was selected for prokaryotic expression. After expanded cultured, the pMD19-T-IFNω5 plasmid was extracted from the above positive transformants. Using it as template and ExIFNω-F/R as primers, PCR was performed to obtain the mature peptide DNA fragment. Then the fragment and pGEX-6P-1 were double digested by *Eco*R I and *Not* I, and the digested products were purified respectively. After that, the 2 enzyme-digested products were ligated to construct the recombinant plasmid pIFNω and it subsequently transformed into competent *E. coli* BL21. Positive transformants were selected with LB solid medium containing 100 μg/mL ampicillin (Amp). Individual colonies were successively identified by PCR, double-enzyme digested, and sequenced.

### Prokaryotic expression, purification and confirmation of rIFNω

For the recombinant IFN-ω protein (rIFNω) induced expression, overnight cultured pIFNω-containing bacteria (rBL21) were added to LB medium (containing 100 μg/mL Amp) at a ratio of 1:100, incubated at 37 °C to OD_600nm_ ≈ 0.6 with the rotate speed of 150 r/min, then 0.1 mmol/L IPTG was added, and continue to cultivate at 16 °C for 4 h. The rBL21 without IPTG induction was set as a control. Meanwhile, the untreated pGEX-6P-1 was transferred into competent *E. coli* BL21 and the same expression procedure was performed as control too. All bacteria were collected by centrifugation. The IPTG-induced rBL21 were resuspended in PBS for ultrasonic crushing to collect the supernatant and pellet. The latter was resuspended in PBS to equal volume. Finally, the collected proteins and control bacteria were electrophoresed at 80 V for 30 min then turn to 120 V and stained by Coomassie brilliant blue for SDS-PAGE analyzing.

For the protein purification, it was used the GST-Tag protein purification Kit. The fluid flow through the column was collected after the addition of bacterial lysate, washing buffer and elution buffer respectively. The ultrafiltration tube with a 10-kDa molecular weight cut-off was used to concentrate protein eluent and remove salts. The liquid obtained from each step was analyzed by SDS-PAGE. Protein concentrations were calculated automatically by absorbance measured at 280 nm (A280) by Nano Drop 2000.

For the rIFNω confirmation, the purified rIFNω and the induced *E. coli* BL21 contained untreated pGEX-6P-1 were performed SDS-PAGE, then transferred to a 0.22-µm nitrocellulose membrane (NCM). The NCM was blocked with 5% skim milk in phosphate-buffered saline containing Tween-20 overnight at 4 °C, and the presence of the GST-tagged proteins was confirmed using anti-GST monoclonal antibody with 2000 times diluted and HRP-conjugated anti-mouse IgG monoclonal antibody with 5000 times diluted. The NCM was developed by BeyoECL Plus and photographed by GelView 6000Plus Intelligent Image Workstation (Biolight, Guangzhou, China).

### Detection of the regulatory activity of rIFNω on ISG transcription

FMD-C1 cells were cultured in DMEM medium with 10% fetal bovine serum at 37 ℃ and 5% CO_2_. The cells (5 × 10^5^ cells/mL, 2 mL/well) were seeded in a 12-well plate, and rIFNω was added to stimulate the cells when they covered 80% of well bottom. For dose–response study: The final concentrations of rIFNω in the medium were 100 ng/mL, 200 ng/mL and 400 ng/mL, respectively. The wells without rIFNω were used as control and incubated. Total RNA was extracted after 6 h and synthesized cDNA. For time-response study: 400 ng/mL rIFNω was added to the medium, and total RNA was extracted after 4 h, 8 h and 10 h of culture, respectively. Wells added rIFNω were extracted immediately used as control for subsequent qPCR.

The above cDNA was used as template, and the primer pairs of ISGs and reference genes were used for qPCR to detect the Ct values of related gene. Three replicates were set for each sample. The reaction volume was 20 μL, including SYBR Green Mix 10 μL, forward and reverse primers (10 pmol/L) 1 μL, cDNA template 1 μL, ddH_2_O 7 μL. The amplification program was 95 ℃ for 3 min, 40 cycles of 95 ℃ for 10 s and annealing 30 s. Followed melting curve analysis was 95 ℃ for 10 s and continuous fluorescent acquisition with heating samples from 65 to 95 ℃.

Excel was used for Ct values processing and analysis. The formula used to calculate the relative transcript level of genes is as follows, where ΔCt = Ct_(control group)_−Ct_(experimental group)_^49^. GraphPad Prism (version 8.0.2) was used to plot and analyze the statistical significance of the data in each group by one-way ANOVA. P < 0.05 was considered significant difference.

$${\text{Relative }}\;{\text{mRNA }}\;{\text{level}} = \frac{{\left( {1 + E_{{{\text{target}}}} } \right)^{{\Delta {\text{Ct }}\;{\text{target}}}} }}{{\frac{{\left( {1 + E_{{{\text{ref1}}}} } \right)^{{\Delta {\text{Ct }}\;{\text{ref}}1}} + \left( {1 + E_{{{\text{ref2}}}} } \right)^{{\Delta {\text{Ct }}\;{\text{ref2}}}} }}{2}}}$$

### Optimization and application of rIFNω-based indirect ELISA

The purified rIFNω protein were diluted to 1 mg/mL with PBS. Following the procedure recommended by QuickAntibody (2W) adjuvant, 6 female mice were injected intramuscularly with the mixture of 50 μg (50 μL) rIFNω protein and equal volume adjuvant, respectively, through the hind leg muscles. After a week, another injection was given in the same way. Blood was collected from the tail vein on day 14 and antibody titers were determined by ELISA. Then the serum of high-titer antibody-producing mice was centrifuged at 3000 g for 5 min to obtain anti-rIFNω polyclonal antibody.

A checkerboard titration was performed to optimize the concentrations of the antigen and antibodies as per the standard protocol. To be specific, 60 μg, 30 μg, 15 μg, 7.5 μg, 3.75 μg, 1.875 μg of rIFNω was coated in 96-well plate, and then the negative serum and positive serum were diluted into 1:50, 1:100, 1:200, 1:400, 1:8, 1:16, 1:32, 1:6400 gradients. According to the pattern of indirect ELISA, 3 parallel replicates of negative and positive sera for each gradient were performed using no protein blocking solution. At 450 nm, the optimum concentration is when the difference between positive and negative absorbance ratio (P/N) is the largest, and the positive value is close to 1 at the same time.

The contents of natural IFNω in serum of 8 forest musk deer were detected by the rIFNω-based indirect ELISA. Undiluted forest musk deer serum was used as antigen and anti-IFNω polyclonal antibody was used as primary antibody. Serum from unimmunized mice was used as negative control.

### Ethics approval

All animal experiments were conducted according to the guidelines for Animal Research: Reporting of In Vivo Experiments and approved by the National Institute of Animal Health Care and the Animal Ethical and Welfare Committee of Sichuan Agricultural University (approval No. SYXK2019-187). All methods were performed in accordance with relevant guidelines and complied with the current laws on animal welfare and research in China.

## Data Availability

The nucleotide and protein sequences obtained during the current study are available in the NCBI repository with the Genbank accession numbers OP331218-OP331222. The rest of the data generated or analysed during this study are included in this published article.
